# Agent-mediated spatial storage effect in heterogeneous habitat stabilizes competitive mouse lemur coexistence in Menabe Central, Western Madagascar

**DOI:** 10.1186/s12898-015-0040-1

**Published:** 2015-03-05

**Authors:** Livia Schäffler, Joachim Saborowski, Peter M Kappeler

**Affiliations:** Behavioral Ecology & Sociobiology Unit, German Primate Center, Göttingen, Germany; Present address: Museum für Naturkunde, Berlin, Germany; Department Ecoinformatics, Biometrics and Forest Growth, and Department Ecosystem Modelling, Büsgen-Institute, Georg-August University of Göttingen, Göttingen, Germany

**Keywords:** Ecological structure, Species assemblage, Interspecific interactions, Competition, Intraguild predation, Agent-mediated coexistence, Spatial storage effect, Lemurs

## Abstract

**Background:**

Spatio-temporal distribution patterns of species in response to natural and anthropogenic drivers provide insight into the ecological processes that determine community composition. We investigated determinants of ecological structure in a species assemblage of 4 closely related primate species of the family Cheirogaleidae (*Microcebus berthae*, *Microcebus murinus, Cheirogaleus medius, Mirza coquereli*) in western Madagascar by extensive line transect surveys across spatial and temporal heterogeneities with the specific goal of elucidating the mechanisms stabilizing competitive coexistence of the two mouse lemur species (*Microcebus* spp.).

**Results:**

Interspecific competition between the mouse lemurs was indicated by negative spatial associations in degraded habitat and by habitat partitioning along anthropogenic disturbance gradients during dry seasons with resource scarcity. In non-degraded habitat, intraguild predator *M. coquereli*, but not *C. medius,* was negatively associated with *M. murinus* on the population level, whereas its regional distribution overlapped spatially with that of *M. berthae*. The species’ interspecific distribution pattern across spatial and temporal heterogeneities corresponded to predictions for agent-mediated coexistence and thus confirmed *M. coquereli*’s stabilizing impact on the coexistence of mouse lemurs.

**Conclusions:**

Interspecific interactions contribute to ecological structure in this cheirogaleid assemblage and determinants vary across spatio-temporal heterogeneities. Coexistence of *Microcebus* spp. is stabilized by an agent-mediated spatial storage effect: *M. coquereli* creates refuges from competition for *M. berthae* in intact habitat, whereas anthropogenic environments provide *M. murinus* with an escape from resource competition and intraguild predation. Species persistence in the assemblage therefore depends on the conservation of habitat content and context that stabilizing mechanisms rely on. Our large-scale population level approach did not allow for considering all potential functional and stochastic drivers of ecological structure, a key limitation that accounts for the large proportion of unexplained variance in our models.

## Background

Understanding the composition of communities, as well as the distribution and relative abundance of their constituent species, i.e. “ecological structure”, has long represented a fundamental question in ecology [1]. Biological interactions have been identified as a major structuring force in taxonomic assemblages: Direct, competitive interactions between species are of particular significance for ecological structure and often result in checkerboard-like distribution patterns, particularly between closely related species [2]. Interspecific predation is a second direct mechanism known to structure ecological communities, which may have even greater effects on the size and distribution of prey populations [3-6]. Intraguild predation (IGP) describes combined effects of competition and predation [7]. Intraguild predators share resources and symmetrically or asymmetrically prey upon each another, with consequences for the distribution and abundance of both species. Coexistence under IGP can be stabilized if the prey represents a superior resource competitor or if the predator gains considerably from prey consumption [8,9].

One member of a species assemblage may also influence pairwise direct interactions between other coexisting species as a “third agent” via competition, predation, or IGP, however. Indirect effects of predation can lead to apparent competition: (intraguild) predation influences the intensity of competition between coexisting prey species, thereby creating spatial patterns similar to those of competitive exclusion [10-12]. Interactions with a third agent may also result in stabilizing coexistence of prey, if species outcompete each other in different tasks: prey superior in resource competition will occur at highest densities in most productive habitats, whereas the species superior in predator avoidance will be found at highest densities in predator-free space [13,14].

Finally, ecological structure also depends on vegetation and species composition of the habitat as well as on the size and distribution of patches that are important for the resident species. Diversity depends on habitat productivity as well as on floristic and structural diversity [15]. Habitat heterogeneity may facilitate coexistence on a regional scale in both, competitive and predator–prey systems [16] if competitive rankings are reversed in patches of different quality, and reciprocal exclusion provides interacting species with refuges from competition or predation (“spatial storage effect” [13,14]). Finally, temporal heterogeneity can promote coexistence of species that share niches by temporal resource partitioning [17]. Because ecological structure is hard to predict, even for very similar communities [18], determinants of ecological structure need to be specifically examined for a given community or taxonomic assemblage.

### Lemur assemblages in Madagascar

Lemurs, the strepsirrhine primates endemic to Madagascar, have been subject to numerous studies of their biogeography and community ecology [19-23] because they are characterized by high α‐diversity and species assemblages comprise many closely related species. Many lemur communities are phylogenetically clustered and predominately structured by environmental conditions [24-26], including Quaternary climatic shifts [23,27,28]. More recently, dramatic rates of habitat destruction and degradation have affected the composition of local lemur communities as well [29]. Susceptibility of lemurs to extinction from fragments increased with body mass and degree of frugivory [30], whereas resilience was promoted primarily by behavioral plasticity [31].

### A cheirogaleid assemblage in Menabe Central

An assemblage of 5 relatively well studied members of the family Cheirogaleidae in the dry deciduous forests of Central Western Madagascar [32] qualifies as a model system to investigate determinants of ecological structure against the backdrop of anthropogenic disturbances producing strong spatial and temporal heterogeneities. These lemurs are all nocturnal, arboreal, relatively small (< 1 kg) and, with one exception (see below), omnivorous.

Madame Berthe’s mouse lemur (*Microcebus berthae*) is the smallest known species of all primates (mean adult mass 31 g [33]). Its range is confined to Menabe Central [34], where it is only found in habitat patches > 30,000 ha [35]. It coexists with the larger gray mouse lemur (*M. murinus*: average adult mass 60 g [36,37], which has a much wider geographic distribution [38]. This species has been observed in all forest types across southern and western Madagascar, including small fragments and the vicinity of villages [35]. The fat-tailed dwarf lemur (*Cheirogaleus medius)* is a larger (mean adult mass: 120 g [39]) dietary generalist that undergoes months of hibernation during the dry cool season [40]. Its geographical range is similar to that of *M. murinus*. Coquerel’s dwarf lemur (*Mirza coquereli*) is a 250 g omnivore found in the forests of central western Madagascar [41,42] that preys upon several species of vertebrates, including other cheirogaleids [43]. Finally, it is unlikely that interactions with the 200 g pale fork-marked lemur (*Phaner pallescens)* are shaping ecological structure of this community, as this species is ecologically differentiated from its family members due to feeding specialization on gum exudates and vertically separated by its habitat use [41,44,45].

Previous studies revealed various interactions among the other four sympatric cheirogaleids. Here, we report on the spatial distribution of coexisting cheirogaleids across *M. berthae*’s biogeographic range. We focus on explaining the distribution and abundance of *M. berthae* because it was only discovered after the pioneering studies by Charles-Dominique et al. [32] and because it is one of the more endangered primates [46]. In addition to interspecific interactions with coexisting cheirogaleids as determinants of ecological structure in this species assemblage, we also consider variation in habitat degradation.

The two *Microcebus* species belong to different subclades [38], indicating allopatric speciation and secondary coexistence after periods of independent history. Considering the differences between the mouse lemurs’ biogeographic ranges as well as population densities, *Microcebus* spp. do not comply with ecological similarity s*ensu* Brown [47]. Moreover, the interspecific body size ratio of *Microcebus* spp. slightly exceeds the Hutchinsonian ratio for “limiting similarity” and therefore does not clearly oppose ecological differentiation [48,49]. Community-wide isotope analysis revealed extensive niche overlap in fruit and animal matter (δ^15^N) between *M. berthae*, *C. medius*, and *M. coquereli*, and in basal resources (δ^13^C) between the two mouse lemurs and *M. coquereli* [50]. Both *Microcebus* spp. are omnivorous and experience similar seasonal fluctuations in food supply [37]. *Microcebus berthae* relies mainly on insect material and its narrow feeding niche is embraced by the wider diet of *M. murinus*, which includes higher amounts of fruit and gum and is subject to greater seasonal variation [51]. The small-scale distribution of *Microcebus* individuals in Kirindy Forest did neither differ in relation to the distribution of homopteran larvae colonies (a key resource during the lean season), nor to preferred sleeping sites [52]. Finally, *C. medius* was found to partially displace *M. murinus* on a local scale, whereas positive spatial associations with *M. berthae* indicated relaxed competition [37].

*Microcebus* spp. are subject to similar top‐down control and face intense predation pressure from several classes of predators, including raptors, snakes, and carnivores [53-55]. Moreover, there is evidence for opportunistic predation by *M. coquereli* on *M. murinus* [43,54,56], but not on *M. berthae*. These intraguild predatory interactions with third agents have not yet been examined as a potential mechanism stabilizing interspecific coexistence of *Microcebus* spp., however.

Based on population assessments across the complete range of *M. berthae*, our study addressed two main questions. First, does the distribution of mouse lemurs indicate competitive interactions? In case of competitive exclusion, we expect negative complementary distributions of the two mouse lemur populations. Second, is coexistence of *Microcebus* spp. stabilized by “third agents”? In this case, we predicted negative complementary distributions of *M. coquereli* and/ or *C. medius* with competitively superior *M. murinus* and spatial overlap with inferior *M. berthae*. In these analyses, we consider both spatial and temporal heterogeneities to account for dependence of competitive and intraguild predatory interactions on resource supply.

## Methods

### Study site

**Figure 1:**
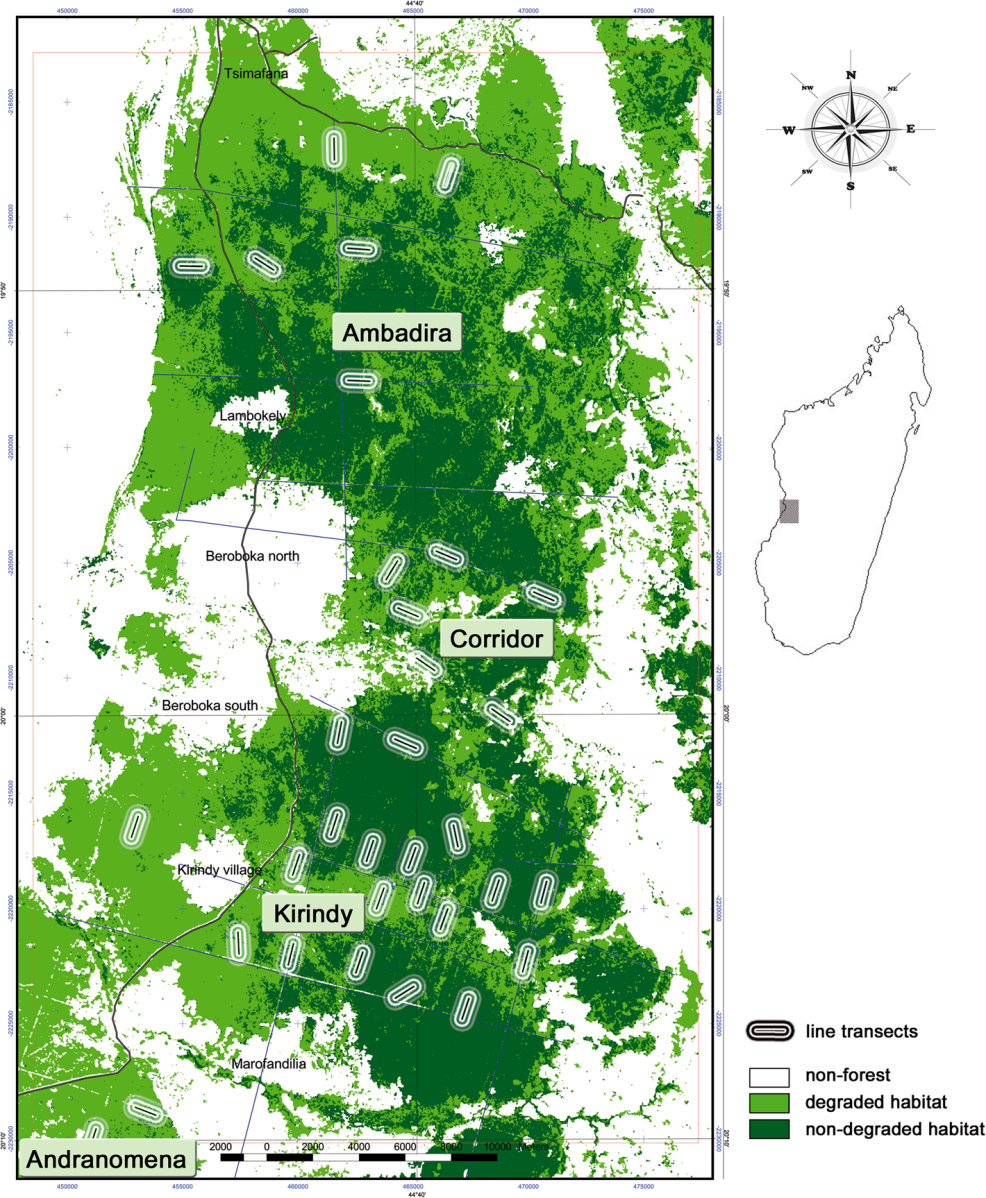
**The study area in central western Madagascar, depicting forest heterogeneity and distribution of line transects across Menabe Central (only two of four line transects shown for RS Andranomena); map based on Landsat 7 ETM 2003, geographic coordinates WGS84, UTM Zone 38.**

The region of Menabe Central comprises the largest remnant of Malagasy dry deciduous forest [57]. The climate in this area between the Tsirihibina and Morondava rivers is characterized by a 4 month hot rainy season (annual mean rainfall 800 mm) and 8 months without precipitation and cool nights (as low as 3°C [58]). Forest cover in this area is being reduced at annual rates of up to 2.5% [59] by slash-and-burn agriculture and logging which have transformed pristine habitat into secondary forest formations, scrub, and savanna [58]. Roads cut into the forest for oil explorations and timber harvesting facilitate public access and anthropogenic activities such as subsistence hunting and the collection of forest products [57,60]. These activities have resulted in three major forest patches of heterogeneous quality connected by degraded forest habitat (Figure 1). Ambadira and Kirindy Forests are connected by a corridor of 5 to 7 km width, and have been increasingly segregated from the Réserve Spéciale Andranomena. Although never effectively protected, Ambadira Forest was only moderately accessed and considerable areas of near primary forest persist [57]. Within Kirindy Forest, illegal activities have been limited by the presence of a forestry concession and a research station [58]. In contrast, RS Andranomena is only legally protected and particularly prone to anthropogenic disturbances due to close proximity of several villages.

### Lemur surveys

We surveyed cheirogaleid species across Menabe Central by repeated transect walks during 4 dry and 2 rainy seasons between 2003 and 2007. To this end, we established 35 1-km line transects, which were evenly distributed to the extent feasible in dense dry deciduous forest; forest areas without abandoned logging trails or oil exploration tracks were often not accessible (Figure 1). In total, we surveyed 34 (Ambadira n = 5, corridor n = 6, Kirindy n = 19, RS Andranomena n = 4) transects during dry season, and 25 (Ambadira n = 4, corridor n = 6, Kirindy n = 11, RS Andranomena n = 4) during rainy season surveys.

During each survey, cheirogaleid populations were appraised on 13–23 transects by line transect walks [61]. *Cheirogaleus medius* was not included in dry season surveys because it spends most of this time in hibernation [40]. The great majority of transects was surveyed twice per survey and several times over subsequent surveys, amounting to a total of 150 1-km samples. In order to control for circadian variation in lemur activity, transect walks were conducted between 6:00 p.m. and 8:30 p.m. on days without rain. Two observers trained to recognize cheirogaleids in their natural habitat at night walked with headlights along line transects at a standardized pace of about 1 km/h. Using torches and binoculars, they identified visually detected individuals to species level independently and approached individuals in great distance from the transect line for identification. In a number of cases where animals could not be identified with confidence sights were recorded as ‘non identified’ and excluded from subsequent analyses [61].

### Ethical note

All research reported in this manuscript is in compliance with animal care regulations and applicable national laws of Germany and Madagascar. All research protocols were approved by the appropriate Animal Use and Care committees of Germany (Bundesamt für Naturschutz, BfN) and Madagascar (Ministère de l’Environment et des Eaux et Forêts, MINEEF).

### Habitat classification

To take differential habitat suitability and disturbance levels into account, we assessed the degradation of the forest surrounding each transect based on stand and understory density, canopy height and cover [61]. In addition, we exemplarily sampled forest structure along 7 transects during the dry season 2007 (3 in non-degraded and 4 in degraded habitat) and along 6 transects during the late rainy season 2008 (4 in non-degraded and 2 in degraded habitat, incl. 1 resample): 21 sampling plots were established every 50 m along 1-km line transects, located alternately on the left and right side 25 m away from the transect. Spatial forest structure was assessed by point-quarter sampling [62]: In each of four quarters per sample point, the distance from the sampling point to the center of the nearest mature tree (DBH ≥ 10 cm) and of the nearest tree older than 10 years (5 cm ≤ DBH < 10 cm) was measured, adding up to 84 (21 plots × 4) trees per size class and transect. For each size class, we compared mean distance of the nearest tree of each class from the center point as a measure of forest density on non-degraded and degraded habitat transects (Mann–Whitney U-test). During the dry season 2007, we additionally recorded regenerating vegetation by counting all trees of 1 cm ≤ DBH < 5 cm within an area of 4 m × 4 m around each sample point and tested tree numbers for differences between non-degraded and degraded habitat. In both assessments, we estimated understory density at every sampling point by systematically positioning a white cloth at breast height in the four compass directions at a distance of 4 meters from the appraiser who estimated visibility in four categories (0-25%, 25-50%, 50-75%, 75-100%). Due to substantial variation in foliage between dry and rainy season, we tested understory density in 2007 and 2008 separately for differences between non-degraded and degraded habitat (Mann–Whitney U-test). Around every sampling point, we visually estimated canopy height in 2007 and tested it for differences between degraded and non-degraded habitat (Mann–Whitney U-test). Canopy cover was estimated by recording whether the open sky was visible through a vertically held pipe at 25 m intervals along transects and respectively 25 m off the trail [35]. As substantial seasonal differences in visibility between seasons prohibited pooling of data, we tested canopy cover sampled in 2007 and 2008 separately for differences between non-degraded and degraded habitat. Finally, we tested variables that significantly differed between degraded and non-degraded habitat for correlations with other forest variables (Spearman rank correlation).

### Data analyses

Analyses were based on encounter rates rather than on density estimates as they are less fraught with assumptions. Density estimates based on line transect survey data rely on accurate assessment of detected individuals’ perpendicular distance from the transect line and is influenced by the selected detection function.

### Pooling of survey data by season

In order to justify pooling of encounter rates per transect over surveys for statistical analyses, we tested specific encounter rates of replicate surveys for differences (F-tests in RBD with transects as blocks). Moreover, cheirogaleid populations show a pronounced postnuptial increase after the midpoint of the rainy season that might prohibit pooling of early and late rainy season data. In order to detect systematic differences between pre-birth and post-birth rainy season, we compared encounter rates for 16 transects sampled during the early and late rainy season (Wilcoxon signed-rank test).

Cheirogaleids’ encounter rates did neither differ significantly between repeated transect walks within single surveys in any of the study regions, nor between replicate surveys within the same season, and they were not influenced by demographic effects [61]. We therefore averaged encounter rates over repeated transect walks and over subsequent surveys. Seasonal differences in detection probabilities as well as in activity patterns (e.g. seasonal torpor in *M. murinus*) were reflected in the encounter rates of *M. coquereli* and in hibernating *C. medius* [61]. In order to allow for documenting cheirogaleids’ responses to temporal variations in food supply, we analyzed dry and rainy season data separately.

### Interspecific distribution of *Microcebus* spp. across heterogeneous habitats

To assess the importance of interspecific competition between mouse lemurs as a function of spatial and temporal heterogeneities, we examined their distribution for potential spatial exclusion from degraded and non-degraded habitat transects in dry and rainy seasons, respectively. For this purpose, we tested mouse lemur encounter rates by season for differences between degraded and non-degraded habitat in which the congener was either present or absent (Mann–Whitney U-test), or occurred in different abundance classes (Kruskal-Wallis ANOVA). Low encounter rates for *M. berthae* only afforded opportunity to use presence/absence as an explanatory factor, whereas encounter rates of abundant *M. murinus* allowed for categorization into four abundance classes (*M. murinus*: absence, ≥ 1 and < 5 ind./km, ≥ 5 and < 10 ind./km, ≥ 10 ind./km).

### Determinants of regional mouse lemur distribution

We fitted log-linear models to the encounter rates of either *Microcebus* spp. in SPSS [63], which allow for appraising the relative strength of structuring factors, as well as for detecting interactions between interspecific effects and environmental variables.

The number of transects was too low to allow for testing all potentially influential variables and factors simultaneously. Therefore, explanatory variables were systematically added based on the results of exploratory analyses [61]. We started adding the forest regions, proxies for anthropogenic disturbances (i.e. habitat degradation and distance to the nearest village), and encounter rates of relevant coexisting cheirogaleids before proceeding to analyzing factor interactions. For each mouse lemur species, we fitted negative binomial distributions of detection events combined with a natural log-link function to logarithmic encounter rates (overdispersion indicated that Poisson models were prone to Type I errors). Encounter rates were corrected by an offset term for varying survey effort (i.e. total transect length). Model fit was assessed based on Akaike’s Information Criterion (lowest AIC/ AICc), and the amount of total variation in encounter rates explained by best-fitting models was quantified by the coefficient of determination (pseudo-R^2^).

## Results

### Habitat classification

We consider our ground-based classification of the forest into non-degraded and degraded habitat reliable, as it was broadly congruent with a forest classification based on a Landsat ETM 7 picture (Figure 1). Non-degraded and degraded habitat did not differ in tree density of any size class (DBH ≥ 10 cm: MWU_7,5_ = 8.0, p = 0.123; 5 cm ≤ DBH < 10 cm: MWU_7,5_ = 11.0, p = 0.291), nor did we detect differences in counts of regenerating trees (1 cm ≤ DBH < 5 cm: MWU_3,4_ = 4.0, p = 0.480) or understory density (dry season: MWU_3,4_ = 2.0, p = 0.150/ rainy season: MWU_4,2_ = 2.0, p = 0.355). Canopy height was higher in non-degraded habitat in the available dry season sample (MWU_3,4_ = 0.0, p = 0.034). Differences in canopy cover only became apparent during the dry season, when it was more closed in non-degraded as in degraded habitat (MWU_3,4_ = 0.0, p = 0.034), whereas rainy season forest cover did not differ between non-degraded and degraded habitat (MWU_4,2_ = 0.0, p = 0.060). However, canopy cover was negatively related to the mean distance of trees from the center point in both size categories (trees older than 10 years with 5 cm ≤ DBH < 10 cm as well as mature trees with DBH ≥ 10 cm: Spearman r = −0.637, n = 12, p = 0.026). Moreover, mean distances from PCQM center points in trees of the two size classes were positively correlated (Spearman r = 0.846, n = 12, p = 0.001). Thus, there are indications that closed canopy cover in non-degraded habitat is associated with higher tree density.

### Interspecific distribution of *Microcebus* spp. across heterogeneous habitats

Complying with habitat partitioning along anthropogenic disturbance gradients, mouse lemurs divergently tracked seasonal changes in carrying capacity in non-degraded and degraded habitat. During the dry season, *M. berthae* was present on 62.5% of non-degraded habitat transects, and on 38.8% of degraded habitat transects. During the rainy season, we encountered *M. berthae* on 72.7% of transects surveyed in non-degraded habitat, but only on 21.4% of those in degraded habitat (for details see [34]). Thus, the population of *M. berthae* spread out to degraded habitat during the dry season, while it concentrated in non-degraded habitat during the rainy season. In contrast, *M. murinus* was encountered on 87.5% of transects surveyed in non-degraded habitat and on 72.2% in degraded habitat during the dry season. During the rainy season, *M. murinus* was present on 45.5% of transects in non-degraded habitat and on 42.9% in degraded habitat (for details see [61]). The species therefore spread out by local dispersal during the dry season across habitats, whereas its population was concentrated on fewer transects during the rainy season in both, intact and degraded habitat.

**Figure 2:**
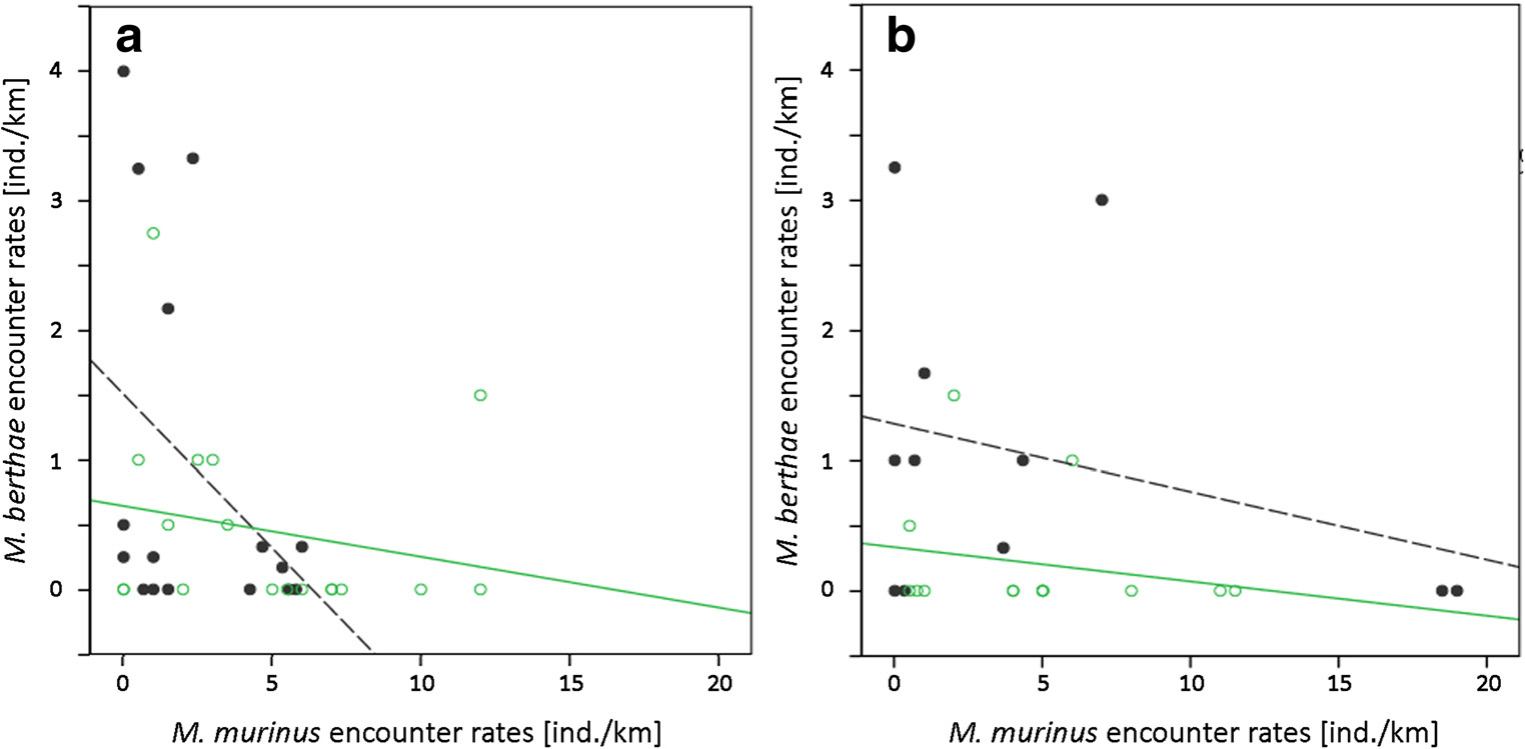
**Encounter rates of** ***M. berthae*** **in [a] dry and [b] rainy season on transects with varying** ***M. murinus*** **encounter rates; black filled points and dashed line: non-degraded habitat, green circles and continuous line: degraded habitat; abundance classes: absent, low (< 5 ind./km), medium (5 ≤ ind./km < 10) and high (**≥ **10 ind./km).**

*Microcebus berthae* encounter rates differed between transects classified by *M. murinus*’ encounter rates only during the dry season in degraded habitat (Kruskal-Wallis H_3,18_ = 9.419, p = 0.024). In the dry season, we did not encounter any *M. berthae* individuals in degraded habitat when *M. murinus* was absent or occurred in medium abundance. *Microcebus berthae*’s encounter rates were highest on transects with low encounter rates of the congener, but the species also occurred on some transect with high *M. murinus* encounter rates (Figure 2a, b). No significant differences in encounter rates appeared in non-degraded habitat during the dry season (H_2,16_ = 2.946, p = 0.229) or, regardless of habitat type, during the rainy season (non-degraded: H_3,11_ = 4.139, p = 0.247; degraded: H_2,14_ = 0.697, p = 0.706). However, Figure 2b indicates that during the rainy season, *M. berthae* only occurred on transects on which *M. murinus* was absent or present in low abundance.

We did not find differences in *M. murinus*’ encounter rates between transects with *M. berthae* either present or absent, regardless of season or habitat degradation (dry season, non-degraded: MWU_6,10_ = 21.0, p = 0.327; dry season, degraded: MWU_11,7_ = 25.5, p = 0.238; rainy season, non-degraded: MWU_4,7_ = 11.0, p = 0.567; rainy season, degraded: MWU_11,3_ = 11.5, p = 0.456).

### Determinants of regional mouse lemur distribution across spatial and temporal heterogeneities

#### Microcebus berthae

**Figure 3:**
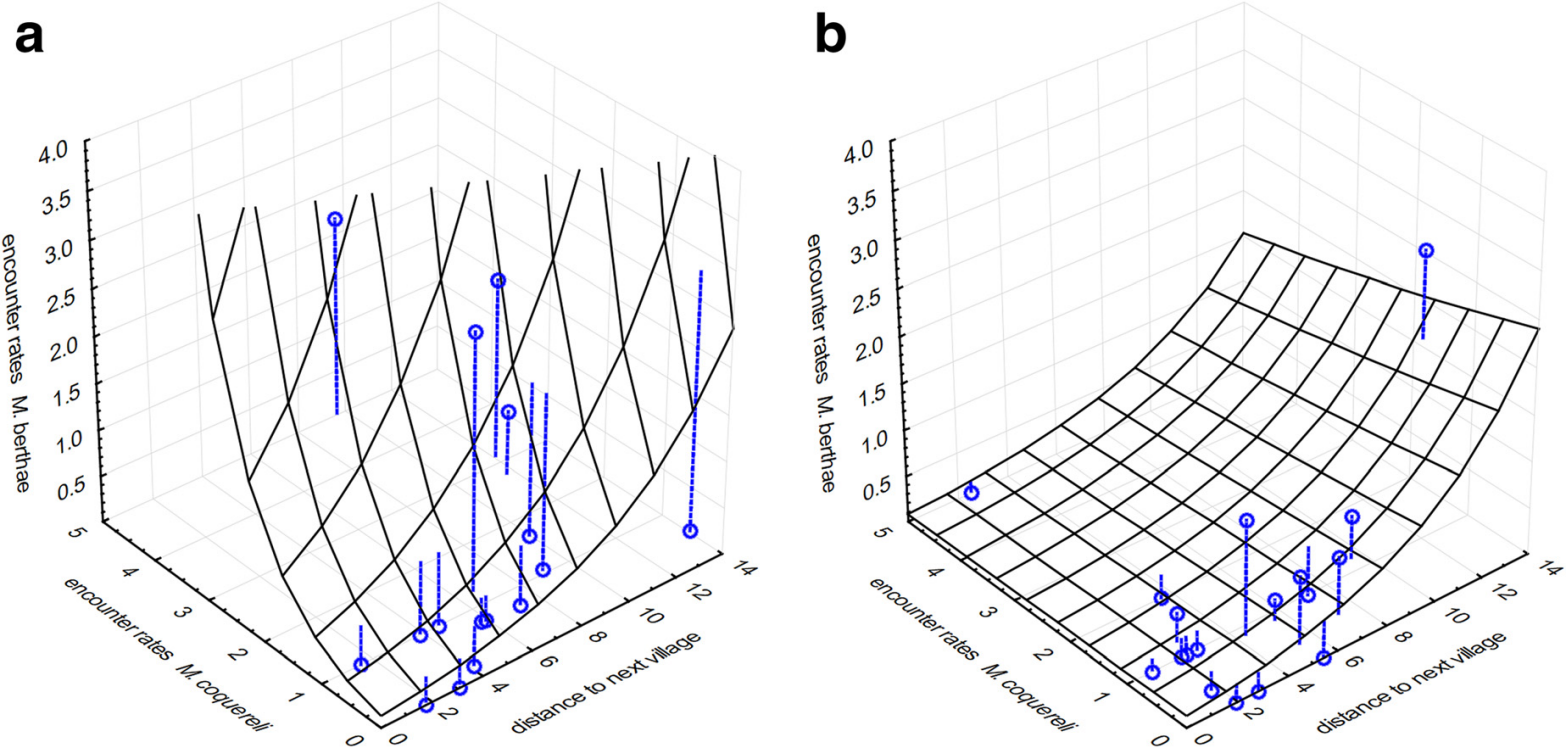
**Observed dry season encounter rates of** ***M. berthae*** (points) and predictions by log-linear model (curved surfaces) in [a] non‐degraded (n = 16, model equation: ER_Mb.ds = exp(−2.075 + 1.148*ER_Mc.ds + 0.211*dist.village)) and [b] degraded habitat (n = 18, model equation: ER_Mb.ds = exp(−2.075-0.09*ER_Mc.ds + 0.211*dist.village)) across Menabe Central; deviance of observed encounter rates from model predictions are represented by dashed lines.

The distribution of *M. berthae* in either season was influenced by habitat degradation. During the dry season, we found a positive association with *M. coquereli* in non-degraded habitat, but not in degraded habitat. Moreover, dry season encounter rates of *M. berthae* significantly rose with increasing distance to the nearest village (Table 1).The log-linear model with the best fit to dry season encounter rates of *M. berthae* explained 10.74% of total variance (Figure 3).

**Table 1:** **Dry season modelling results for** ***M. berthae***

**n (transects)**	**Survey effort [km]**	**n (obs)**	**AIC**	**AICc**	***pseudo-R*** ^***2***^
34	96	69	116.070	117.45	10.74%
**Dry season model coefficients**		**B**	**df**	**p**	
Constant term		−2.075			
Factor interactions	ER_Mc.ds in non-degraded habitat	1.148	1	0.019*	
	ER_Mc.ds in degraded habitat	−0.09	1	0.845	
Distance to nearest village		0.211	1	0.016*	

^(^*^)^, Significance level 0.1 > p ≥ 0.05; *, significance level 0.05 > p ≥ 0.01; **, significance level p < 0.01.

**Figure 4:**
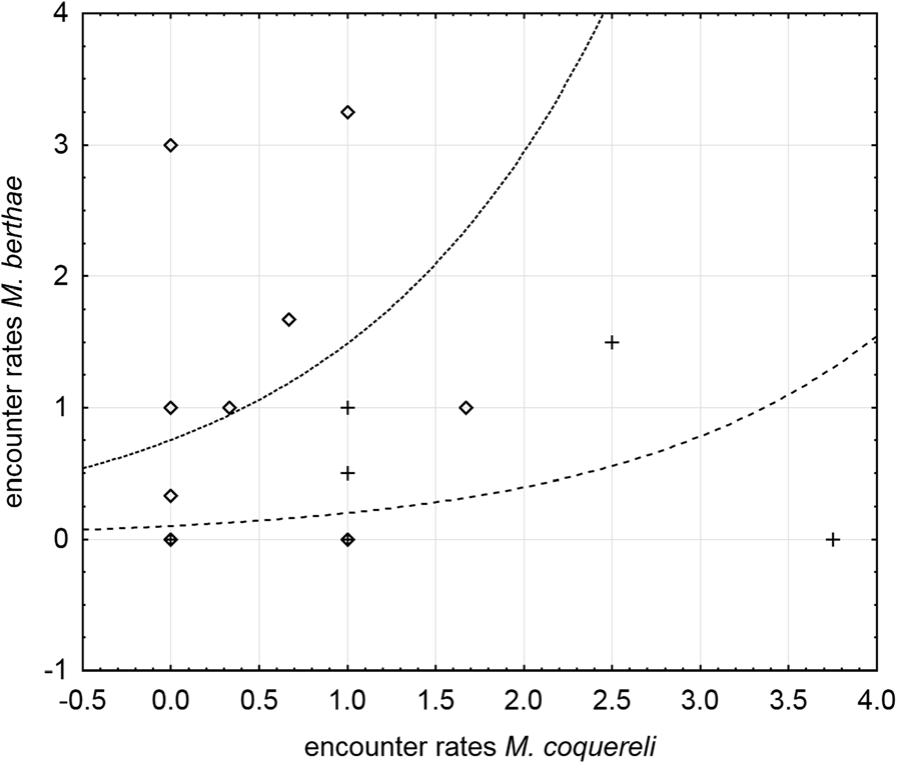
**Observed rainy season encounter rates of** ***M. berthae*** (squares: non-degraded habitat, crosses: degraded habitat) and predictions by log-linear model (dot-dash fine line: non-degraded habitat, model equation: ER_Mb.rs = exp(−2.289 + 2.007 + 0.681*ER_Mc.rs); dot-dash rough line: degraded habitat, model equation: ER_Mb.rs = exp(−2.289 + 0.681*ER_Mc.rs)) across Menabe Central; due to low variance in ***M. coquereli*** **rainy season encounter rates, model predictions of numerous transects overlap (only 5 different encounter rate values in non‐degraded (n = 11) and 4 in degraded habitat (n = 14)).**

During the rainy season, we encountered more *M. berthae* in non-degraded than in degraded habitat. The positive association of *M. berthae* with *M. coquereli* was independent of habitat degradation, but non-significant, and the population distribution was not related to the distance from villages (Table 2). The best fitting log-linear model accounted for 13.05% of total variance in rainy season encounter rates (Figure 4).

**Table 2:** **Rainy season modelling results for** ***M. berthae***

**n (transects)**	**Survey effort [km]**	**n (obs)**	**AIC**	**AICc**	***pseudo-R*** ^***2***^
25	56	40	73.826	74.969	13.05%
**Rainy season model coefficients**		**B**	**df**	**p**	
Constant term		−2.289			
Non-degraded habitat		2.007	1	0.005**	
Degraded habitat		0			
ER_Mc.rs		0.681	1	0.094^(^*^)^	

^(^*^)^, Significance level 0.1 > p ≥ 0.05; *, significance level 0.05 > p ≥ 0.01; **, significance level p < 0.01.

#### Microcebus murinus

**Figure 5:**
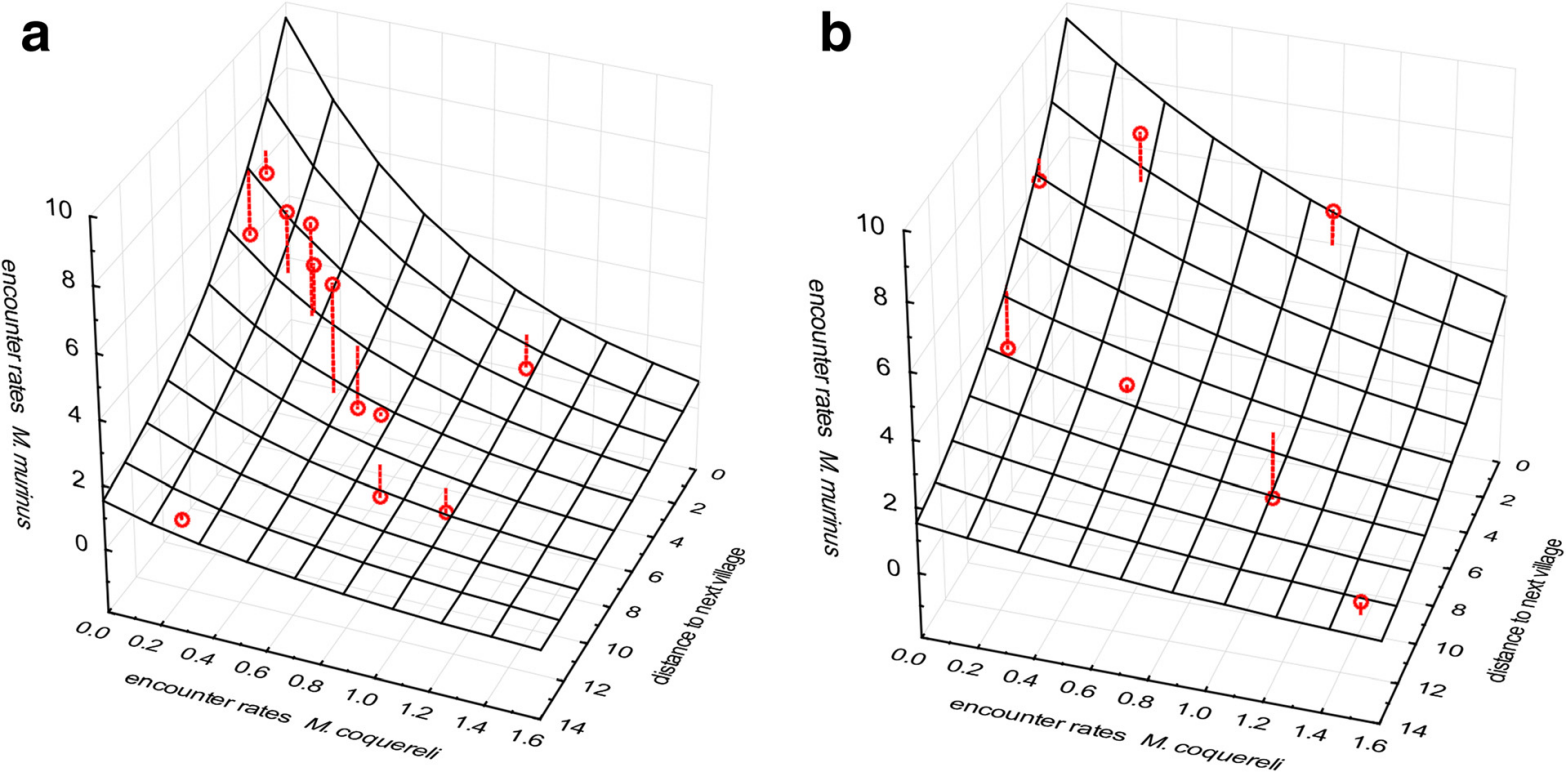
**Observed dry season encounter rates of** ***M. murinus*** (points) and predictions by log-linear model (curved surfaces) in [a] non‐degraded (n = 12, model equation: ER_Mm.ds = exp(3.130-0.876-1.479*ER_Mc.ds-0.131*dist.village) and [b] degraded habitat (n = 7, model equation: ER_Mm.ds = exp(3.130-0.876-0.675*ER_Mc.ds-0.131*dist.village) within Kirindy Forest; deviance of observed encounter rates from model predictions are represented by dashed lines.

During the dry season, *M. murinus’* distribution varied regionally, with significantly lower encounter rates in Ambadira compared to other forests. Moreover, *M. murinus* was negatively associated with *M. coquereli* in non-degraded habitat, and encounter rates decreased non-significantly with increasing distance from a village (Table 3). In order to enhance comprehensibility, model predictions and observations are only shown for Kirindy Forest in Figure 5. The dry season model explained 10.25% of total variance in *M. murinus*’ encounter rates.

**Table 3:** **Dry season modelling results for** ***M. murinus***

**n (transects)**	**Survey effort [km]**	**n (obs)**	**AIC**	**AICc**	***pseudo-R*** ^***2***^
34	96	347	212.273	216.581	10.25%
**Dry season model coefficients**		**B**	**df**	**p**	
Constant term		3.130			
Region	Ambadira	−2.117	1	0.021*	
	Corridor	−0.413	1	0.614	
	Kirindy	−0.876	1	0.249	
	RS Andranomena	0			
Factor interactions	ER_Mc.ds in non-degraded habitat	−1.479	1	0.023*	
	ER_Mc.ds in degraded habitat	−0.675	1	0.165	
Distance to nearest village		−0.131	1	0.079^(^*^)^	

^(^*^)^, Significance level 0.1 > p ≥ 0.05; *, significance level 0.05 > p ≥ 0.01; **, significance level p < 0.01.

**Figure 6:**
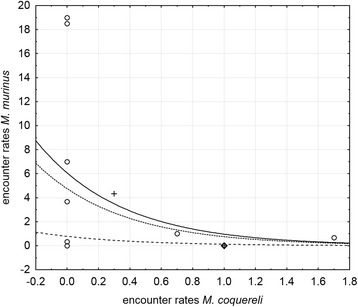
**Observed rainy season encounter rates of** ***M. murinus*** **(squares: Ambadira Forest, crosses: corridor, circles: Kirindy Forest) and predictions by log-linear model (dot-dash rough line: Ambadira Forest, dot-dash fine line: corridor, continuous line: Kirindy Forest) in non‐degraded habitat (n = 11) across Menabe Central; due to low variance in** ***M. coquereli*** rainy season encounter rates, model predictions of numerous transects overlap (only 5 different encounter rate values in non‐degraded habitat); RS Andranomena not represented as it entirely consists of degraded habitat; model equations for non-degraded habitat in Ambarida Forest: ER_Mm.rs = exp(1.878-2.128-1.841*ER_Mc.rs), in the corridor: ER_Mm.rs = exp(1.878-0.320-1.841*ER_Mc.rs), in Kirindy Forest: ER_Mm.rs = exp(1.878-0.077-1.841*ER_Mc.rs).

Rainy season data yielded a negative interspecific association with *M. coquereli* in non-degraded habitat (Figure 6). Regional variation in the distribution of *M. murinus* across forest regions was non-significant, and vicinity to villages did not have any effect. Rainy season predictions were based on coefficients given in Table 4 and the model term accounted for 9.11% of total variance.

**Table 4:** **Rainy season modelling results for** ***M. murinus***

**n (transects)**	**Survey effort [km]**	**n (obs)**	**AIC**	**AICc**	***pseudo-R*** ^***2***^
25	56	229	162.932	167.599	9.11%
**Rainy season model coefficients**		**B**	**df**	**p**	
Constant term		1.878			
Region	Ambadira	−2.128	1	0.096^(^*^)^	
	Corridor	−0.320	1	0.644	
	Kirindy	−0.077	1	0.902	
	RS Andranomena	0			
Factor interactions	ER_Mc.rs in non-degraded habitat	−1.841	1	0.004**	
	ER_Mc.rs in degraded habitat	0.021	1	0.964	

^(^*^)^, Significance level 0.1 > p ≥ 0.05; *, significance level 0.05 > p ≥ 0.01; **, significance level p < 0.01.

## Discussion

The mouse lemurs’ regional distribution across spatio-temporal heterogeneities was negatively complementary and indicated habitat partitioning when resources were scarce and where coexistence stabilizing mechanisms were lacking. Interspecific distribution of the mouse lemur species in relation to *M. coquereli* complied with predictions derived from the hypothesis of a third agent’s coexistence stabilizing impact: In either season, log-linear models suggested that *M. coquereli* regulates *M. murinus’* abundance predominately in non-degraded habitat, which *M. berthae* essentially relies on [34], whereas *M. berthae*’s regional distribution largely matched that of *M. coquereli*. In contrast, we found no consequences of interspecific interactions with *C. medius* for the spatial population structure of mouse lemurs.

### Direct interspecific interactions: interspecific competition in *Microcebus* spp.

Local movements of mammalian dietary specialists to track resources in seasonally dry tropical forests are widespread [64], and there is evidence that community dynamics change in response to localized resource heterogeneity [65]. Thus, a spatial storage effect in a permanent spatio-temporally heterogeneous environment represents a plausible mechanism facilitating coexistence in *Microcebus* spp.: the two mouse lemurs are relieved from intense interspecific competition in heterogeneous habitat types at different times of the year, and competitive exclusion is prevented by retreat into the respective refuges.

During the dry season, *M. berthae*’s expansion to degraded habitat may be a consequence of resource tracking along forest edges, where homopteran larvae aggregate [66]. Yet, the species evaded anthropogenic environments at this time of the year, when increased forest accessibility may favor human frequentation [57]. Concentration in non-degraded habitat during the rainy season complies with most pronounced productivity increases in intact forest. During the dry season, the population of *M. murinus* was more dispersed than during the rainy season, and positive spatial association with villages was only vaguely indicated. Anthropogenic environments may provide *M. murinus* with exclusive resources and relax interspecific competition with *M. berthae* in degraded habitat at a greater distance from villages. Alternatively, *M. murinus* might be excluded from forest edges at a greater distance from villages by interspecific interactions and be crowded into anthropogenic environments that are not suitable for sympatric species. Behavioral observations of interspecific interactions at feeding sites suggested feeding priority of *M. murinus* [52], making spatial exclusion by direct interactions with *M. berthae* therefore unlikely. Absence from some degraded habitat transects during the dry season is in line with the finding that suitability of degraded habitat is limited even for disturbance-tolerant *M. murinus*: the capacity to enter daily torpor is constrained by fewer resting holes and higher ambient temperatures in secondary habitat, and individuals have lower body mass and higher mortality risk than in primary forest [67]. Occurrence of *M. murinus* in high abundances on some non-degraded habitat transects that was limited to the rainy season is likely a result of capacity tracking [68].

We can rule out two alternative explanations for the divergent results from the rainy and the dry season: First, lack of seasonal differences in *Microcebus* encounter rates challenges the interpretation that the pattern is simply caused by differential activity patterns, particularly due to dry season inactivity in *M. murinus* females. Second, rainy season foliage should hamper visibility more drastically in non-degraded than in degraded habitat. However, during the rainy season we encountered more *M. berthae* individuals and the species was present on more transects in non-degraded habitat; *M. murinus* encounter rates were higher during the rainy season on some transects in non-degraded habitat.

### Indirect interspecific interactions: agent-mediated coexistence

Our log-linear models incorporating the distribution of sympatric cheirogaleids did not reveal direct negative interspecific associations of *Microcebus* populations on the regional scale, but pointed towards a more complex coexistence stabilizing mechanism, such as a third agent’s stabilizing impact on the competitive coexistence of *Microcebus* spp. Habitat selecting predators often stabilize interspecific coexistence by forcing prey into certain habitat types [4], if landscape heterogeneity provides species with refuges [9]. Interspecific interactions with *M. coquereli* appeared to enhance spatial heterogeneity and contribute to stabilizing coexistence in *Microcebus* spp. by an agent-mediated spatial storage effect [14,68]. *Microcebus berthae* benefitted from relaxed competition with the congener in non-degraded habitat, whereas *M. murinus* escaped negative interspecific interactions with the third agent by crowding into anthropogenic environments [69]. Thus, the observed negative complementary dry season distribution of mouse lemurs in relation to the distance from villages rather corresponds to apparent competition than to habitat partitioning along disturbance gradients, i.e. it represents an agent-mediated indirect interaction [10]. Alternatively, lack of the third agent’s regulating impact on *M. murinus*’ population structure in anthropogenic environments during the dry season might locally release this species from IGP pressure and favor direct interspecific local exclusion of *M. berthae*.

Predator-mediated coexistence is considered an important factor for maintaining diversity in many natural communities [70-73] that has been acknowledged since Paine’s pioneering study [3] on the diversity in rocky intertidal communities: the sea star *Pisaster ochraceus* preys upon two competing sessile mussel species (*Mytilus californianus* and *M. trossulus*) and prevents exclusion of the inferior competitor by preferential predation on the stronger competitor (but see also [74]). Competitors that share a common predator have been found to be distributed along various environmental stress gradients in many natural systems (e.g. freshwater communities in temporary ponds [75]).

The pattern observed in this cheirogaleid assemblage complies with predictions of species interaction models involving two species that share resources and a common predator and outcompete each other in the different tasks [76,77]: Predators facilitate interspecific coexistence of prey predominately at intermediate levels of productivity with intermediate predation risk, as observed for the mouse lemurs during the dry season in non-degraded habitat and during the rainy season in degraded habitat. By contrast, the superior competitor dominates at low resource supply (and low predation risk), a prediction that was met during the dry season by the negative spatial association of the mouse lemurs in degraded habitat and by the largely exclusive occupancy of anthropogenic environments by *M. murinus*. At high productivity levels and high predation risk, the predator-resistant inferior competitor dominates, corresponding to the aggregation of *M. berthae* in non-degraded habitat during the rainy season, where it spatially overlapped with the population of *M. coquereli*. Opportunistic predation on *M. murinus* in non-degraded habitat during the dry season therefore represents a plausible determinant of ecological structure. During the rainy season, *M. coquereli*’s regulative impact on *M. murinus* population may alternatively be attributed to intensified feeding competition among capacity-tracking cheirogaleids in productive habitat. This would comply with extensive niche overlap in basal resources between the mouse lemurs and *M. coquereli* [50]. However, competition alone cannot explain the observed interspecific distribution pattern, as *M. berthae* is not competitively excluded by *M. coquereli* although the two species are isotopically indistinguishable in fruit and animal matter as well as in basal resources. We therefore conclude that *M. coquereli* can be attributed the role of a “keystone (intraguild) predator” that controls the abundance of a primary consumer by preferential predation and/ or resource competition, which in turn is capable of excluding other species from the community (for the key-stone species concept see [78]).

Proposed mechanisms of predator-mediated coexistence include predator preference switching to the most common prey, predators preferring the dominant competitor, and predators altering the resources used by competing prey, thereby affecting competition between them [79]. Predator‐mediated coexistence of *Microcebus* spp. was excluded earlier due to assumed similarity and the same seasonal variations in predation risk [37], but in particular as *M. berthae*’s mortality rates exceeded that of superior competitor *M. murinus* [52]. However, given the great variety of potential anti‐predator strategies and the high selection pressure, mouse lemurs may likely have evolved divergent behavioral responses and therefore be differentially affected by specific (intraguild) predators [80]. Moreover, predators usually favor one particular species among a set of potential prey, either via specific preference or density dependence [10,13], and reduce the relative abundance of the preferred prey [81]. Even if predation risk is shared among prey, negative effects of predation on a species can be coupled with an indirect positive effect of a competitor being consumed [82,83].

Mouse lemur coexistence can be stabilized via opportunistic predation by *M. coquereli* irrespective of the question whether the prey species is selected by specific preference or in a density‐dependent manner: Predation has the potential to stabilize coexistence among prey species if it is directed preferentially towards the most frequent by preventing competitive exclusion [70,84]. In case of density‐dependent predation, *M. coquereli* would also capture disproportionally more *M. murinus* individuals, which are easier to locate as they occur in higher densities [61] and are clumped in space [51,85]. Differential predation or competitive pressure exerted by *M. coquereli* on the two mouse lemur species is still to be shown by behavioral studies. However, IGP does not only operate via predatory interactions, but also via interspecific competition, and effects of an intraguild predator on the ecological structure in assemblages of closely related species are therefore rather likely.

We did not find indications for *C. medius* acting as a third agent on a regional scale to shape the spatial population structure of *Microcebus* populations. Lack of positive spatial association between *M. berthae* and *C. medius* on the population level may be due to differential microhabitat preferences [37]. Interspecific spatial exclusion of *M. murinus* despite overlapping ecological requirements might be prevented by this mouse lemur’s acceptance of habitats unsuitable to *C. medius*, which therefore represent competitor-free rainy season refuges. Our data do not allow for drawing conclusions on the situation during the dry season, but hibernating *C. medius* presumably reduce the number of sleeping holes available for *M. murinus* to some extent.

### Community composition and system stability in view of habitat change

The ecological structure in the cheirogaleid assemblage arises from two general niche-based processes that determine the structure of many communities: convergence of coexisting species due to environmental filtering (trait‐based assembly rules [86]) and divergence resulting from interspecific interactions (niche-based assembly rules [77]). The closely related species comprising the cheirogaleid assemblage are more similar than expected from random assortment, most likely in consequence of overlapping ecological requirements as in many natural communities (mammals [87], isopods [88], dyscids [89]). On the other hand, we observed consequences of interspecific interactions on the spatial population structure of the mouse lemurs, which have been identified as a major structuring force in many taxonomic assemblages (trees [90], desert rodents [91-93], pond snails [94], tadpoles [95]).

Our results emphasize the importance of habitat quality and heterogeneity for system stability. Low productivity promotes competitive exclusion and hampers the coexistence of ecologically similar species [91]. In line with theoretical knowledge [76,77], α‐diversity in lemurs is highest at medium disturbance levels [96] and drops with increasing agricultural intensity [97]. Accordingly, this cheirogaleid assemblage was deprived of one or more species where anthropogenic pressure is particularly intense. For example, the species particularly susceptible to anthropogenic disturbances were common in Ambadira, but largely absent from RS Andranomena. As in many natural systems [12,75,98-103], such habitat heterogeneity provides cheirogaleid species with refuges from detrimental interspecific interactions and allows for coexistence on a regional scale. White‐footed mice in a fragmented landscape, for example, were released from interspecific competition with larger granivores in smaller patches, whereas they were excluded from larger patches [104].

Changes in habitat content and context affect different species in different ways and alter the structure of communities [31,105,106]. Habitat reduction alters the level of interspecific competition and predation pressure [107-111], and anthropogenic habitat fragmentation affects specialist species more severely than generalists [112]. Within isolated subpopulations, competitive pressure can increase to a level that causes local interspecific exclusion [113] and the effects that predators exert on prey populations may aggravate. If fragmentation hampers recolonization of suitable patches, local extinction can be irreversible [30]. Occurrence of North American gray squirrels in an agriculturally fragmented landscape was positively related to the size of remaining fragments, whereas sympatric red squirrels could only persist in patches providing particular resources and were excluded from patches occupied by gray squirrels due to increased competition [114]; fragmentation additionally prevented (re-)colonization of isolated patches and consequently affected community structure in some squirrel species [115].

Populations do not necessarily respond linearly to habitat loss and fragmentation, but can decline abruptly over a narrow range of habitat impairment when extinction thresholds are exceeded [116]. Given the complexity of ecological communities, any species’ removal or addition will have indirect effects on multiple levels [83]. Loss of a single species, even if originally rare, may trigger an extinction cascade that potentially extends to a large number of species and therefore may have dramatic consequences for community stability [117]. Extinction of predators intensifies the impact of habitat loss on regional abundance of prey species, which are consequently threatened with local extinction [118]. Particularly incidental prey for generalist predators were found more imperiled than the predators by habitat degradation and to face the greatest risk of extinction [119]. Consequences of keystone predator losses are exemplified by the destruction of macrophyte associations due to increased herbivory by sea urchins after removal of sea otters [120], or by the displacement of San Joaquin kit foxes following population increases in coyotes after local extinction of North American wolves [121].

Given that interspecific interactions in heterogeneous habitat shape the ecological structure of our cheirogaleid assemblage, system stability essentially depends on the preservation of habitat content and context. In view of the ongoing population decline in third agent *M. coquereli* across the species’ entire range (more than 50% over a period of 10 years), the species was recently rated as endangered [122]. On a regional scale, extensive population fluctuations in *M. coquereli* [123] are compensated by immigration from adjacent populations [124], but continuing fragmentation may put *M. coquereli* at risk of extinction from patches that are too remote to allow for recolonization. As predator removal will have the strongest effect on species in trophic levels beneath it when the prey are most extensively engaged in competition [125], loss of *M. coquereli* would likely corrupt ecological structure in the cheirogaleid assemblage and ultimately drive *M. berthae* to extinction. Thus, conservation of remaining high quality habitats as well as retaining their connectivity will be crucial to prevent biodiversity loss in Menabe Central.

## Conclusions

Our results hint at the complexity of factors determining ecological structure in this small primate assemblage, including the mechanism stabilizing mouse lemur coexistence, which depend on both spatial and temporal habitat heterogeneity. On a methodological note, the spatial scale of this study justifies the phenomenological measure of abundance, even if it may not be the most appropriate way to characterize how species respond to habitat heterogeneity: treating populations as entities does neither account for the major components of population change [126], nor for divergent reactions of individuals and age‐specific behaviors [127], which can fundamentally change interspecific interactions and the likelihood for spatial exclusion [9]. Finally, the best models left a large proportion of the total variation in measures of abundance unexplained. A key limitation of the species-oriented approach is that not all important variables can be included in analyses of ecological structure [126]. In order to extend our approach from the assemblage to the community level, additional variables should be incorporated in multivariate analyses, such as floristic diversity [96] or the distribution of key resources [52]. Finally, neutral assembly processes (ecological drift and dispersal limitation) need to be considered in order to assess the relative contribution of stochastic and deterministic drivers to ecological community structure [24,128].

## Abbreviations

IGP: Intraguild predation
RS: Réserve Spéciale
δ^13^C: Isotopic signature: delta-C-13 is a measure of the ratio of stable isotopes ^13^C:^12^C [‰]
δ^15^N: Isotopic signature: delta-N-15 is a measure of the ratio of ^15^N:^14^N [‰]
SPSS: Software package for statistical analysis
DBH: Diameter at breast height
RBD: Randomized Block Design
AIC: Akaike’s Information Criterion
AICc: AIC with a correction for finite sample sizes
pseudo-R^2^: Coefficient of determination
B: Log-linear model coefficient
df: Degrees of freedom
ind.: Individuals
ER: Encounter rate [ind./km]
Mb: *Microcebus berthae*
Mm: *Microcebus murinus*
Mc: *Mirza coquereli*
ds: Dry season
rs: Rainy season
dist.village: Distance to the nearest village
dist.village: Distance to the nearest village
^(^*^)^: significance level 0.1 > p ≥ 0.05; *, significance level 0.05 > p ≥ 0.01; **, significance level p < 0.01
DPZ: German Primate Center GmbH
PCI: Primate Conservation, Inc.
CI: Conservation International
DWTC: Durrell Wildlife Conservation Trust
CAFF/CORE: committee approving research in Madagascar, consisting of representatives of Madagascar National Parks, the Ministry of the Environment, Forests and Tourism (MEFT), and the Ministry of Higher Education
ANGAP: Malagasy National Association for the Management of Protected Areas
